# Spatially restricted subcellular Ca^2+^ signaling downstream of store-operated calcium entry encoded by a cortical tunneling mechanism

**DOI:** 10.1038/s41598-018-29562-9

**Published:** 2018-07-25

**Authors:** Raphael Courjaret, Maya Dib, Khaled Machaca

**Affiliations:** Department of Physiology and Biophysics, Weill Cornell Medicine Qatar, Education City – Qatar Foundation, Doha, Qatar

## Abstract

Agonist-dependent Ca^2+^ mobilization results in Ca^2+^ store depletion and Store-Operated Calcium Entry (SOCE), which is spatially restricted to microdomains defined by cortical ER – plasma membrane contact sites (MCS). However, some Ca^2+^-dependent effectors that localize away from SOCE microdomains, are activated downstream of SOCE by mechanisms that remain obscure. One mechanism proposed initially in acinar cells and termed Ca^2+^ tunneling, mediates the uptake of Ca^2+^ flowing through SOCE into the ER followed by release at distal sites through IP_3_ receptors. Here we show that Ca^2+^ tunneling encodes exquisite specificity downstream of SOCE signal by dissecting the sensitivity and dependence of multiple effectors in HeLa cells. While mitochondria readily perceive Ca^2+^ release when stores are full, SOCE shows little effect in raising mitochondrial Ca^2+^, and Ca^2+^-tunneling is completely inefficient. In contrast, gK_Ca_ displays a similar sensitivity to Ca^2+^ release and tunneling, while the activation of NFAT1 is selectively responsive to SOCE and not to Ca^2+^ release. These results show that in contrast to the previously described long-range Ca^2+^ tunneling, in non-specialized HeLa cells this mechanism mediates spatially restricted Ca^2+^ rise within the cortical region of the cell to activate a specific subset of effectors.

## Introduction

Ca^2+^ is a ubiquitous intracellular messenger that encodes a plethora of cellular functions in response to agonists or environmental stimuli. These include contraction, gene transcription, secretion, cellular proliferation, and apoptosis to name a few. Given the disparate often conflicting (cell death *vs* proliferation for example) cellular functions determined by Ca^2+^ signals, cells have developed mechanisms to encode specific cellular responses in the spatial, temporal and amplitude dynamics of Ca^2+^ signals. Spatial encoding can be enforced by co-localization of the Ca^2+^ source (typically a Ca^2+^ channel) and the target effector. Ca^2+^ diffusion around the Ca^2+^ source is restricted by physical and biochemical barriers, creating a space and time limited microdomain where Ca^2+^ can effectively and specifically activate a target. The microdomain can range from a few nm (nanodomains) to a couple of µm depending on multiple factors, including but not limited to channel density and conductance and buffering capacity in the microdomain^1^. Stimulating a Ca^2+^ sensitive mechanism far from the main Ca^2+^ source is consequently a signaling challenge since Ca^2+^ needs to be carried to the effector while avoiding non-specific activation of Ca^2+^ effectors along the way^2^.

Ca^2+^-linked agonists mobilize store Ca^2+^ release typically through activation of IP_3_-Receptors (IP_3_Rs), leading to store depletion and to the opening of Store-Operated Ca^2+^-Entry (SOCE) channels at the plasma membrane (PM). SOCE activation in response to ER store depletion, is mediated by the ER transmembrane Ca^2+^ sensors of the STIM family (STIM1 and STIM2 and their splice variants), which undergo a conformational change in response to reduced luminal Ca^2+^ concentrations, leading to their clustering at ER-PM contact sites (MCS), and recruitment and gating of the PM Ca^2+^ channels of the Orai family (Orai1, 2 and 3)^3^. Ca^2+^ flowing through Orai1 channels is subsequently taken up into the ER by the Sarcoplasmic Endoplasmic Reticulum ATPase (SERCA) pump, thus refilling Ca^2+^ stores and preparing the cell for another round of signaling. However, in addition to store refilling SOCE specifically activates multiple downstream effectors^3–5^. A limitation in that regard is the spatial spread of the SOCE Ca^2+^ microdomain. The assembly of STIM and Orai is not diffuse at the plasma membrane, but highly localized to ER-PM MCS where the two proteins form dense clusters visible at the plasma membrane plane and enhanced in size when SOCE elements are overexpressed^6^. Accumulating evidence suggests that these clusters are not restricted to a simple STIM/Orai partnership but contain numerous additional proteins, including the SERCA pump. Several studies report that, upon store depletion, SERCA localizes to the SOCE clusters^5–10^. This increases the efficiency of Ca^2+^ store refilling by placing the pump close to SOCE Ca^2+^ entry source. This particular organization of the SOCE complex defines a spatial Ca^2+^ microdomain that limits the spread of Ca^2+^ ions entering through Orai1 to a limited sub-membrane area^6^. The actual size of this domain is still a matter of debate. The cell type, the extent of store depletion and the physiological state of the cell will generate various cluster sizes. In addition, experimentally STIM-Orai clusters have been measured following overexpression, which is likely to influence their size^6^. Nonetheless, the most accurate estimates of SOCE clusters come from thin sections studies where the maximal diameter is around 300 nm^11,12^. These clusters are also tightly confined in the “z-axis” due to the close apposition of the ER and PM to create a “disc-shaped” microdomain with a 10–20 nm in thickness^6,13^. This means that for downstream targets to be specifically responsive to SOCE they have to localize in this spatially restricted and cluttered microdomain. This is the case for adenylate cyclase 8 and for the Ca^2+^-activated phosphatase calcineurin, which couples to Orai1^14,15^.

Another mechanism to transport Ca^2+^ in the long-range and expand the SOCE Ca^2+^ microdomain that has been well described in polarized pancreatic acinar cells and *Xenopus* oocytes is Ca^2+^ tunneling^7,16^. Ca^2+^ tunneling involves the transport of Ca^2+^ ions that flow through SOCE channels into the ER lumen, followed by their release through IP_3_ receptors at distal sites to activate effectors without inducing a global Ca^2+^ rise (see recent review^2^). Here we extend the characterization of Ca^2+^ tunneling and show that it encodes exquisite specificity in activating effectors downstream of SOCE. Using real time simultaneous imaging of Ca^2+^ in the ER lumen, cytosol and mitochondria, we show that Ca^2+^ tunneling differentially signals to downstream effectors. Whereas, Ca^2+^ influx through Orai1 within the SOCE microdomain effectively activates NFAT translocation, Ca^2+^ tunneling expands the spatial spread of SOCE by activating other PM effectors such as Ca^2+^-activated potassium channels. Surprisingly however, tunneling is unable to mediate a Ca^2+^ rise in the mitochondria arguing that in the non-polarized HeLa cell it results in a spatially restricted cytosolic Ca^2+^ rise in the cell cortex. This indicates that the long range tunneling mechanism described in polarized cells has been adapted to a preferentially cortical Ca^2+^ signaling module downstream of SOCE allowing selective activation of Ca^2+^-dependent effectors.

## Results

### Simultaneous real time Ca^2+^ imaging in the cytosol, ER and mitochondria

We initially sought to determine the response of mitochondria to Ca^2+^ tunneling downstream of SOCE. Conceptually mitochondria represent an ideal target for Ca^2+^ tunneling, as they form close membrane contact sites with the ER (Mitochondria Associated Membranes or MAMs) where IP_3_ receptors are enriched^17^. Furthermore, the mitochondria are a major target of Ca^2+^ signaling, and play a critical role in buffering cytosolic Ca^2+^ transients. The relationship between SOCE and the mitochondria is complex and exhibits some cell specific nuances. In immune cells the mitochondria have been shown to act as a Ca^2+^ sink for Ca^2+^ flowing through SOCE, which in turn modulates its function^18–20^. The mitochondria is further well established as a low pass filter for cytosolic Ca^2+^ signals and have been shown to functionally interact with SOCE and modulates its signaling^21^. Furthermore, the subcellular localization of mitochondria affects their response differentially to the source of Ca^2+^. In both HeLa cells and pancreatic acinar cells the source of Ca^2+^ was shown to differentially stimulate different populations of mitochondria based on subcellular localization^22,23^. In HeLa cells mitochondria localize away from the plasma membrane^24^, making them an interesting distal target for Ca^2+^ tunneling.

**Figure 1:**
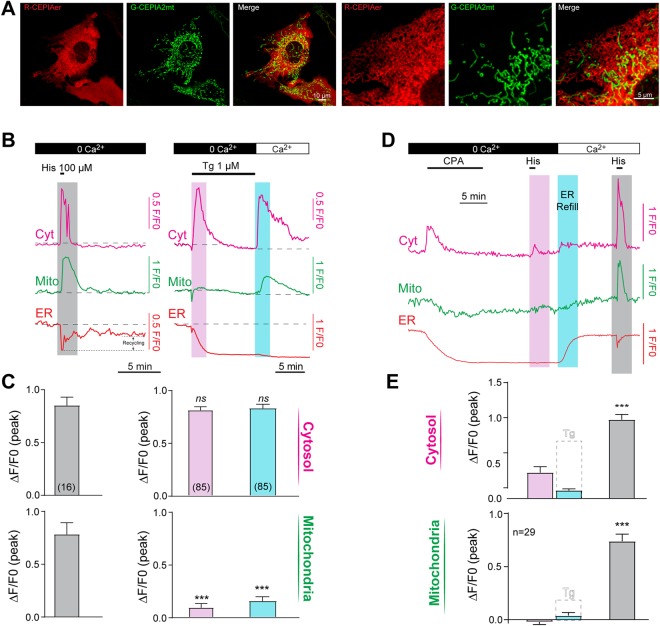
Monitoring Ca^2+^ in the cytosol, ER and mitochondria simultaneously. (**A**) Expression pattern of the ER Ca^2+^ sensor R-CEPIAer and the mitochondria Ca^2+^ sensor G-CEPIA2mt expressed in HeLa cells. (**B**) Variations in Ca^2+^ levels in the cytosol (cyt), mitochondria (Mito), and ER during store depletion induced by histamine (His, grey shading) and thapsigargin in a Ca^2+^-free media (Tg, pink shading), and when SOCE is allowed by the re-addition of Ca^2+^ (2 mM, blue shading). Cytosolic Ca^2+^ was monitored using Fura-Red, the mitochondria using the fluorescence signal of G-CEPIA2mt, and the ER using R-CEPIAer. Left and right panels come from two different sets of experiments. (**C**) Bar chart summarizing the variations in Ca^2+^ levels in the cytosol and mitochondria during the protocols illustrated in A. **(D)** Ca^2+^ dynamics in response to the reversible SERCA blocker, cyclopiazonic acid (CPA). Cells were exposed to CPA (50 µM) for 10 min in Ca^2+^-free media, and then CPA was washed away for 10 min to allow the SERCA pumps to recover their function. Histamine was then applied (100 µM, 30 s) (pink shading), which failed to generate a large Ca_c_^2+^ signal. Bringing back Ca^2+^ in the extracellular media (blue shading), replenishes ER stores and restores the responsiveness to histamine (grey shading). (**E**) Bar chart summarizing the variations in Ca^2+^ in the cytosol and in the mitochondria during the protocol illustrated in panel D. For reference, the Ca_c_^2+^ and Ca_m_^2+^ signal in response to SOCE induced with thapsigargin (panel C) are also illustrated (dotted lines). Values are given as means ± S.E.M, the number of cells analyzed are indicated on the charts. Statistics are calculated according to Student’s t-test, ANOVA and Tukey’s multiple comparisons test.

To test whether the mitochondria act as an effective downstream effector of Ca^2+^ tunneling, we imaged Ca^2+^ dynamics simultaneously in the mitochondria, ER lumen and cytosol. A recently developed family of Ca^2+^ sensors termed CEPIAs (Ca^2+^-measuring organelle-Entrapped Protein Indicators), can be targeted to the ER or to the mitochondria, and allow simultaneous imaging of the two compartments^25^. We thus combined R-CEPIAer (ER lumen Ca^2+^ sensor) with G-CEPIA2mt (mitochondrial Ca^2+^ sensor) to image Ca^2+^ changes in the ER and mitochondria respectively, because their spectral properties allow for imaging cytosolic Ca^2+^ as well using Fura-red (Fig. 1). Confocal images reveal a reticular pattern for R-CEPIAer and a more perinuclear and filamentous expression for G-CEPIA2mt (Fig. 1A), consistent with distribution of ER and mitochondria in HeLa cells^25–27^.

Mobilizing Ca^2+^ stores with histamine (100 µM, 30 sec) results in a simultaneous rise in Ca_c_^2+^ and Ca_m_^2+^ showing that mitochondria readily detect Ca^2+^ released from stores when the stores are full. Ca^2+^ release was coupled to a transient depletion of ER stores which refill due to do recycling of released Ca^2+^ (Fig. 1B). However, ER refilling was incomplete due to the absence of extracellular Ca^2+^ and thus SOCE, resulting in a sustained low level depletion state of the ER (Fig. 1B), which could be reversed by adding Ca^2+^ to the media (not shown). The relative efficiency of the SERCA pump in capturing released Ca^2+^ in the absence of Ca^2+^ influx was variable from cell to cell. As indicated in Fig. 1B, the ER could partly recover from depletion without the contribution of SOCE. The recaptured amount of Ca^2+^ released in the absence of extracellular Ca^2+^ varied between 7% to 65% with a mean value of 30.1 ± 5.1% (given as a percentage of the reduction in ER signal and measured before extracellular Ca^2+^ re-addition) (Fig. 1B).

We then used the classical SOCE protocol of irreversible inhibition of SERCA with thapsigargin (TG, 1 µM) in Ca^2+^-free solution followed by Ca^2+^ addition to maximally activate SOCE and temporally isolate it. TG results in Ca^2+^ release coupled to emptying of ER Ca^2+^ stores but little to no increase in mitochondrial Ca^2+^ (Ca_m_^2+^) (Fig. 1B). Replenishing extracellular Ca^2+^ results in cytosolic Ca^2+^ influx through SOCE that was not associated with store refilling since SERCA in blocked (Fig. 1B). It should be noted that the mitochondrial Ca^2+^ response was quite variable under this experimental paradigm, with only 56.5% of the cells showing a small mitochondrial Ca^2+^ transient in response to TG-dependent Ca^2+^ release; and 49.5% of cells responding to SOCE (n = 85 cells). The rise in Ca^2+^ recorded in the mitochondria was still significant in both cases (Fig. 1C). This confirms previous studies in HeLa cells showing limited coupling between the SOCE microdomain and the mitochondria^23,24^.

The peak amplitude of the global cytosolic Ca^2+^ signal was similar in response to Ca^2+^ release induced by histamine or thapsigargin, or following maximal SOCE induction (Fig. 1C). The mitochondria though responds in a dramatically distinct way to these similar cytosolic Ca^2+^ transients. Whereas mitochondria responded readily to the histamine induced Ca^2+^ rise, they responded poorly if at all to thapsigargin-induced Ca^2+^ release or SOCE (Fig. 1C). This argues for the existence of spatially defined Ca^2+^ microdomains, not detectable by current Ca^2+^ imaging approaches, but readily sensed by different effectors. Mitochondria could be taking up Ca^2+^ released from the ER directly through MAMs when the stores are full. To assess whether this is the case, we recorded at higher scanning speed (1 Hz) to determine the temporal relationship between Ca_c_^2+^ and Ca_m_^2+^. The rise in Ca_m_^2+^ consistently followed the Ca_c_^2+^ transient with a significant delay (3.4 ± 0.2 s, n = 54) (Supplemental Fig. 1), similar to what was reported previously^28^.

The SOCE signal recorded with thapsigargin is maximal and non-physiological, since thapsigargin irreversibly blocks SERCA and prevents store refilling. To obtain a better measure of physiological SOCE, we used the reversible SERCA inhibitor cyclopiazonic acid (CPA). CPA induced a store depletion and a Ca_c_^2+^ rise similar to thapsigargin (61 ± 2% for Tg, n = 85 and 56 ± 3%, for CPA n = 29), without any significant increase in Ca_m_^2+^ (Fig. 1D,E). The peak cytosolic Ca^2+^ release induced by CPA was slightly smaller than that induced by thapsigargin (0.64 ± 0.04 *vs* 0.80 ± 0.03, p < 0.05). Exposing the cell to histamine after CPA treatment in Ca^2+^-free solution resulted in a significantly reduced Ca^2+^ release (Fig. 1D,E, pink bar), showing that CPA efficiently depletes Ca^2+^ stores. When extracellular Ca^2+^ was reintroduced, it allowed quick refilling of the stores (Fig. 1D, blue bar), and a detectable but very small SOCE as a rise in Ca_c_^2+^ (Fig. 1D,E, blue bar). The active refilling of the stores shows that CPA was washed out effectively and that SERCA is functional. The SOCE-dependent cytosolic Ca^2+^ transient detected under this experimental paradigm is orders of magnitude smaller than that detected with TG, yet it efficiently refills Ca^2+^ stores (Fig. 1D,E), arguing that SERCA is effective at limiting Ca^2+^ diffusion outside the SOCE microdomain into the bulk cytosol by sequestering Ca^2+^ flowing through SOCE channels into the ER lumen. Consistent with this interpretation, SOCE under these conditions does not induce any mitochondrial Ca^2+^ rise (Fig. 1D,E). When histamine was applied after reloading of the ER stores, it elicited a large rise in Ca_c_^2+^ and Ca_m_^2+^ indicating normal function of the Ca^2+^ signaling machinery and no deleterious effects of the experimental protocol (Fig. 1D,E, grey bar).

### Mitochondria do not respond to Ca^2+^ tunneling

**Figure 2:**
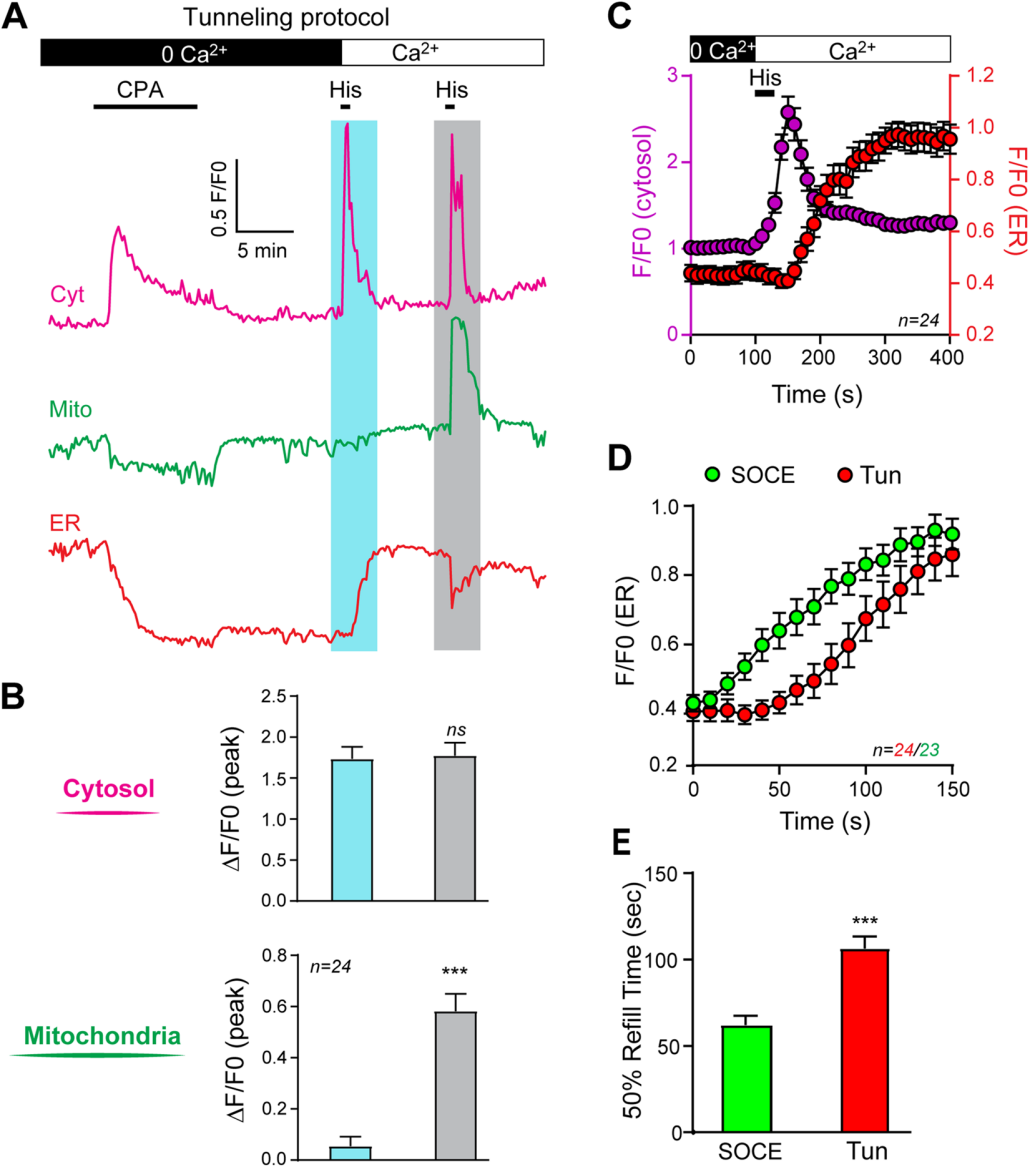
Mitochondria do not respond to Ca^2+^ tunneling. (**A**) To temporally isolate Ca^2+^ tunneling, Ca^2+^ stores were depleted using CPA, followed by a wash out of CPA, and then histamine (100 µM, 30 s) was applied together with extracellular Ca^2+^ (blue shading). This induces Ca^2+^ entry through SOCE, with SERCA active and open IP_3_Rs to allow for Ca^2+^ tunneling. When the ER stores have regained their original level, histamine is applied again (grey shading). (**B**) Bar chart summarizing the variations in Ca^2+^ levels in the cytosol and mitochondria during the protocols illustrated in panel A. (**C**) Average time course of the cytosolic Ca^2+^ signal (purple) and of the ER Ca^2+^ signal (red) during Ca^2+^ tunneling. (**D**) Comparative time course of the refill kinetics of the ER during Ca^2+^ tunneling (red) as compared to during physiological SOCE (green, as in Fig. 1D blue bar). (**E**) Quantification of the refill time (measured at 50% of the maximum refill) during physiological SOCE and during tunneling. Data are means ± S.E.M, statistics are performed using a paired Student’s t-test, the number of cells is indicated in each panel.

The above data show that the mitochondria in HeLa cells do not respond to Ca^2+^ entry through SOCE. However, the experimental conditions above using CPA followed by a wash do not result in the opening of IP_3_ receptor and as such would not allow for Ca^2+^ tunneling as would be expected in response to agonist stimulation. To directly test whether the mitochondria act as a downstream effector of Ca^2+^ tunneling, we devised a protocol to temporally isolate Ca^2+^ tunneling from the Ca^2+^ release phase. We depleted Ca^2+^ stores with CPA (10 min) followed by a wash to release SERCA from inhibition in Ca^2+^-free conditions (Fig. 2A). This was followed by the addition of histamine (100 µM, 30 s) concurrently with extracellular Ca^2+^ re-addition, thus opening IP_3_ receptor and allowing for Ca^2+^ tunneling (Fig. 2A). Surprisingly, histamine and Ca^2+^ application extracellularly under these conditions results in a large cytosol Ca^2+^ rise of similar amplitude to that induce by histamine when Ca^2+^ stores are full (Fig. 2A,B). Under these conditions, histamine cannot induce Ca^2+^ release since the stores are empty due to the CPA treatment (see Fig. 1D, pink bar). Therefore, the large Ca^2+^ transient observed under these conditions requires that Ca^2+^ entering the cell through SOCE be first pumped into the ER by SERCA and then released through IP_3_R, i.e. the classical tunneling pathway. To confirm that our experimental approach induces Ca^2+^ tunneling, we analyzed the time course of the Ca_c_^2+^ rise due to tunneling as compared to ER refilling. As shown in Fig. 2C, the Ca_c_^2+^ signal due to Ca^2+^ tunneling precedes the initiation of ER refilling by tens of seconds, a phase during which Ca^2+^ flowing through SOCE is preferentially taken up into ER stores and release again through IP_3_R to expand the spatial spread of SOCE. Presumably, the large conductance of IP_3_Rs prevents store refilling during this phase. As IP_3_ is metabolized IP_3_Rs close allowing SERCA to refill the stores (Fig. 2C). To confirm our interpretation that during Ca^2+^ tunneling Ca^2+^ leak through IP_3_ receptors delays store refilling, we superimposed the time course of ER refilling during Ca^2+^ tunneling as in Fig. 2A (blue bar) with that during Ca^2+^ refilling in the absence of histamine as in Fig. 1D (blue bar). There is a clear statistically significant (p < 0.001) delay in store refilling during tunneling where the stores require 106.4 + 7.0 sec to reach half-maximal filling compared to 62.1 + 5.3 sec in the absence of histamine (Fig. 2D,E).

Surprisingly though and despite its large amplitude, the Ca_c_^2+^ rise due to tunneling did not produce a Ca^2+^ rise in the mitochondria (Fig. 2A,B; blue bar). However, when the stores where replenished, a second identical application of histamine resulted in a large rise in Ca_m_^2+^ (Fig. 2A,B, grey bar). This shows that Ca^2+^ signals generated by Ca^2+^ tunneling downstream of SOCE or by agonist-dependent mobilization when Ca^2+^ stores are full are not equivalent in terms of inducing a Ca^2+^ response in the mitochondria despite the fact that they result in an equivalent Ca_c_^2+^ rise and are both mediated through IP_3_ receptors. Therefore, Ca^2+^ tunneling and Ca^2+^ release are distinct in their ability to activate downstream effectors. These results argue that the coupling between Ca^2+^ influx through SOCE, uptake by SERCA and release into the cytosol when Ca^2+^ tunneling is operational is highly efficient at raising cytosolic Ca^2+^ in a spatially localized fashion as it is not detected by mitochondria.

We further confirmed that Ca^2+^ flowing through SOCE is not limiting in terms of producing a Ca_m_^2+^ response in these experiments by raising extracellular Ca^2+^ to 10 mM during the tunneling protocol to increase Ca^2+^ flow through SOCE. This results in a similar cytoplasmic signal and did not restore the mitochondrial signal (Supplemental Fig. 2). Another prediction from the Ca^2+^ tunneling pathway is that it would be slower in mediating the Ca_c_^2+^ rise as compared to Ca^2+^ release on full stores. The tunnel mechanism requires two more steps as compared with Ca^2+^ release: first Ca^2+^ influx through SOCE channels and second uptake in the ER lumen by SERCA, independently of a potential delay required for the diffusion of Ca^2+^ into the ER cisternae. It was not possible to resolve this potential delay in the imaging experiments of the three compartments simultaneously because of the slow sampling speed (0.1 Hz). To allow a higher speed of recording (1 Hz), we loaded the cells solely with the Ca^2+^ indicator Fluo4-AM and performed the same protocol as in Fig. 2. The analysis of the time course using either a “virtual” line scan or a global measurement of the Ca_c_^2+^ over time shows a significantly slower rise in the Ca_c_^2+^ mediated by Ca^2+^ tunneling as compared to Ca^2+^ release on full stores, although both signals ultimately reached similar amplitudes (Supplemental Fig. 3).

Another formal possibility in mediating the mitochondrial Ca^2+^ respond to Ca^2+^ release as compared to Ca^2+^ tunneling is the spatial localization of the mitochondria in relationship to the Ca^2+^ source. Depending on the cell type (and physiological conditions) the mitochondria can be positioned close to the plasma membrane as in immune cells where they regulate SOCE^19,20^ or deeper in the cell where they preferentially interact with the ER^23^. We therefore tested whether store depletion affects the relative position of mitochondria in HeLa cells. However, we could not detect changes in mitochondrial localization using either confocal or TIRF imaging (Supplemental Fig. 4). We also evaluated the changes in the morphology of the mitochondria after store depletion using 3D imaging, although we detected some reduction (9%) in the length of mitochondria branches the effect was small and irreversible after Ca^2+^ readdition, and therefore unlikely to explain the differential effect of Ca^2+^ and Ca^2+^ tunneling on mitochondria (Supplemental Fig. 5).

Taken together our results reveal that given the spatio-temporal dynamics of Ca^2+^ release versus Ca^2+^ tunneling, and despite the fact that both rely on IP_3_Rs and are associated with an equivalent rise in global Ca_c_^2+^, mitochondria respond readily to Ca^2+^ release but not Ca^2+^ tunneling. We hypothesize that the slower speed of Ca_c_^2+^ rise during tunneling, combined with the distance between the point source of Ca^2+^ entry (the Orai channel) and the target (i.e. the mitochondria) impair the formation of a “Hot Spot” or high Ca^2+^ domain between the ER and the mitochondria that would allow the MCU to import Ca^2+^ during tunneling.

### Ca^2+^-activated potassium channels

**Figure 3:**
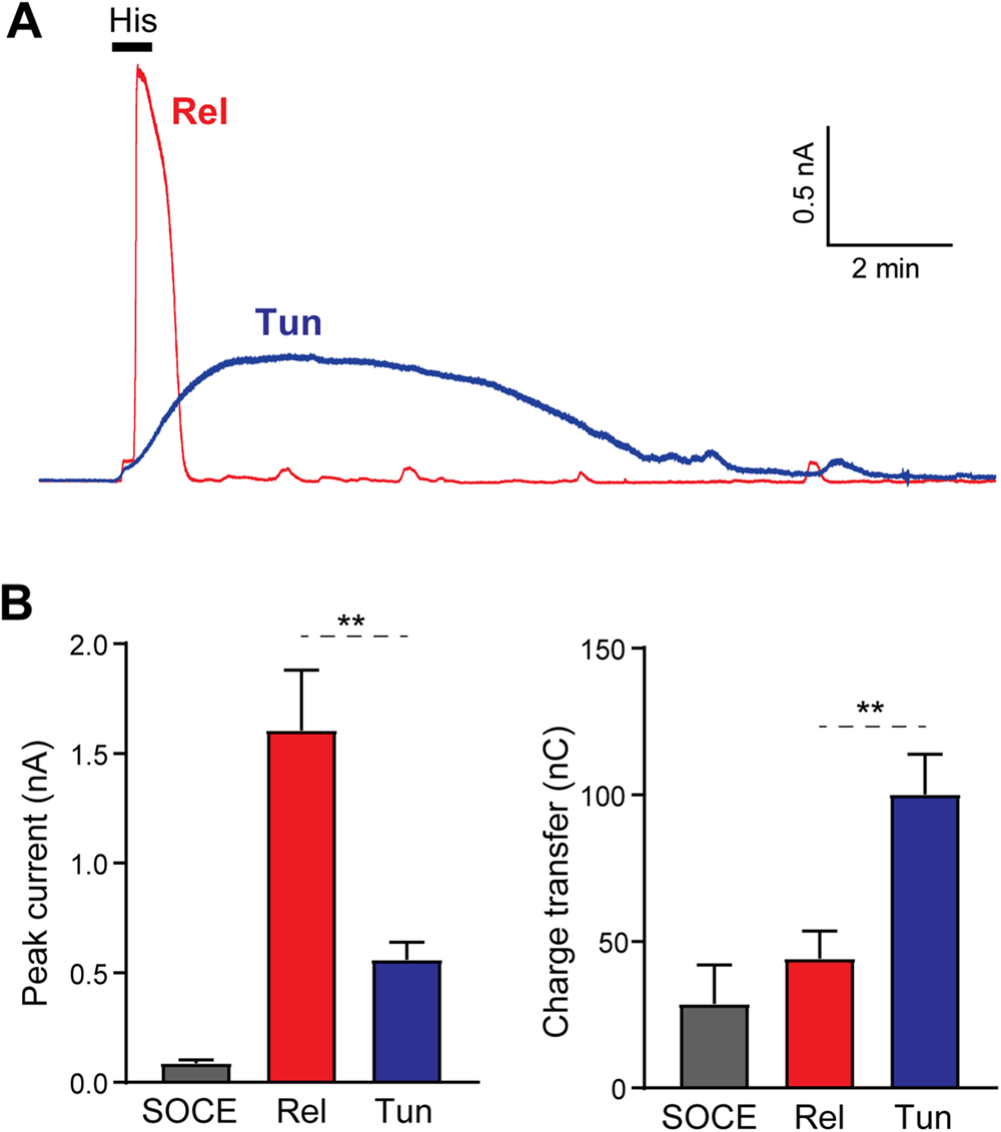
Ca^2+^-activated K^+^ channels in response to Ca^2+^ tunneling and Ca^2+^ release. (**A**) Cells were voltage-clamped in the whole-cell configuration at 0 mV to enhance the driving force for K^+^. The bath application of histamine (100 µM, 30 s) in a Ca^2+^-free media induced a transient outward current. When Ca^2+^ tunneling is induced a slow developing outward current is observed. (**B**) Bar charts summarizing the current amplitude and charge transfer (summed over a 5 min period) obtained in response to SOCE (after CPA treatment), Ca^2+^ tunneling (Tun), Ca^2+^ release on full stores (Rel). Statistics are according to Student’s unpaired t-test.

The global Ca^2+^ rise detected following tunneling argues for a spatially localized cytosolic Ca^2+^ transient. The mitochondria, which tend to localized deeper within HeLa cells, are unable to detect this transient. In *Xenopus* oocytes Ca^2+^ tunneling is particularly effective at stimulating Ca^2+^-activated Cl channels at the PM^7^. Therefore, to test whether Ca^2+^-dependent PM localized effectors respond to Ca^2+^  tunneling, we turned to Ca^2+^-activated K^+^ channels (K_Ca_), which are expressed in HeLa cells^29^. Histamine-dependent Ca^2+^ release when stores are full induces a transient K_Ca_ current (Fig. 3A and Supplemental Fig. 6). Similarly, Ca^2+^ tunneling was effective at activating K_Ca_ (Fig. 3A,B, Tun), but with a smaller peak amplitude than the current induced by histamine from full stores (Fig. 3A,B, Rel). The tunneling-induce K_Ca_ was longer lasting though, leading to a larger charge transfer for an identical histamine stimulation (Fig. 3B). Comparatively, a much smaller maximal gK_Ca_ current was observed in response to SOCE, using the CPA-wash protocol (Fig. 3B, SOCE). This indicates that Ca^2+^ tunneling activates gK_Ca_ at the PM, significantly more efficiently than SOCE (Fig. 3B), in a similar fashion to what is observed with Ca^2+^-activated Cl channels^7^. This is consistent with data from human submandibular gland cells where gK_Ca_ activation by Ca^2+^ influx required the uptake of Ca^2+^ in the ER stores^30^.

**Figure 4:**
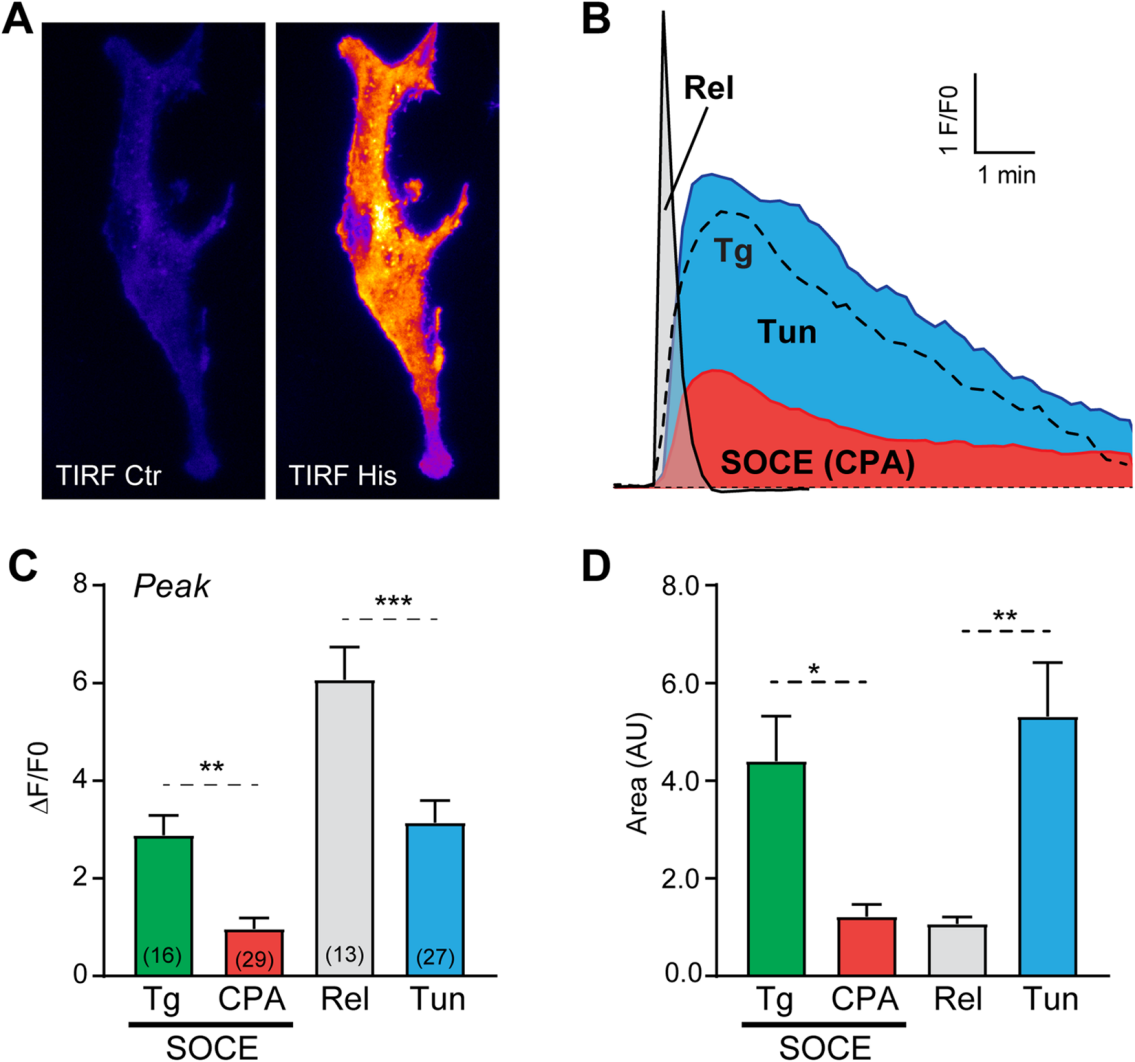
TIRF imaging of Ca^2+^ dynamics in the cell cortex using Lck-GCamp5G. (**A**) Representative TIRF images of the fluorescence of the plasma membrane-anchored Ca^2+^ sensor Lck-GCamp5G at rest (Ctr) and during application of histamine (His; 100 µM, 30 s). (**B**) Typical examples of the time course of the different Ca^2+^ signals recorded under the plasma membrane following: Ca^2+^ release from full Ca^2+^ stores with Histamine (Release); Ca^2+^ tunneling with SOCE re-fueling the ER and releasing Ca^2+^ through IP_3_Rs stimulated by histamine (Tunnel); and SOCE induced by CPA after washout or after store depletion by thapsigargin (Tg). (**C**) Bar chart summarizing the peak amplitude of the SOCE signals induced after store depletion with either thapsigargin or CPA, and that induced following Ca^2+^ release and in response to Ca^2+^ tunneling. (**D**) To account for the difference in the signal kinetics, the area under the trace was integrated over a 5 min period and summarized in a bar char. The number of cells is indicated above the bars, statistics are according to ANOVA followed by Tukey’s multiple comparison test.

The differential response of gK_Ca_ and the mitochondria argue that Ca^2+^ tunneling is effective at raising cytosolic Ca^2+^ levels in the cell cortex close to the PM but not deep within the cell. To record gK_Ca_ we used the whole-cell patch-clamp, which modifies the intracellular environment after breaking into the cell and could have deleterious effects on Ca^2+^ buffering and spatial dynamics. We therefore sought a non-invasive approach to visualize cortical Ca^2+^ transients and confirm the results obtained with gK_Ca_. We used a membrane targeted Ca^2+^-sensor Lck-GCamp5G^31^, coupled to TIRF microscopy to measure Ca^2+^ changes specifically at plasma membrane level. Bath application of histamine induced a fast transient elevation of Ca^2+^ in the sub-PM layer (Fig. 4A,B). Comparatively, Ca^2+^ influx through ‘physiological’ SOCE activated using the CPA-washout protocol results in a smaller, slower and longer lasting cortical Ca^2+^ increase (Fig. 4B). In contrast, when SOCE was induced maximally with thapsigargin it results in larger cortical Ca^2+^ influx (Fig. 4B,C), thus confirming that irreversible inhibition of SERCA enhances cortical Ca^2+^ transients. In agreement with the gK_Ca_ data, Ca^2+^ tunneling results in a slow and long lasting increase in sub-PM Ca^2+^ of significantly higher amplitude and duration then SOCE (Fig. 4B,C). In a pattern similar to what we observed with gK_Ca_ the amplitude of the tunneling signal was smaller than the release induced by histamine on full stores, but significantly longer lasting, creating a larger transfer of total Ca^2+^ (evaluated using the area under the Ca^2+^ traces) (Fig. 4D). Interestingly, during tunneling the total amount of Ca^2+^ ions in the cell cortex is equivalent to that observed with thapsigargin-induced SOCE (Fig. 4D). This highlights the efficiency of the pump-leak pathway at the ER membrane during tunneling, and the conversion of the slow conductance of SOCE channels into a sustained signal at the sub-PM using the high-conductance leak of IP_3_ receptors.

### NFAT1 translocation

**Figure 5:**
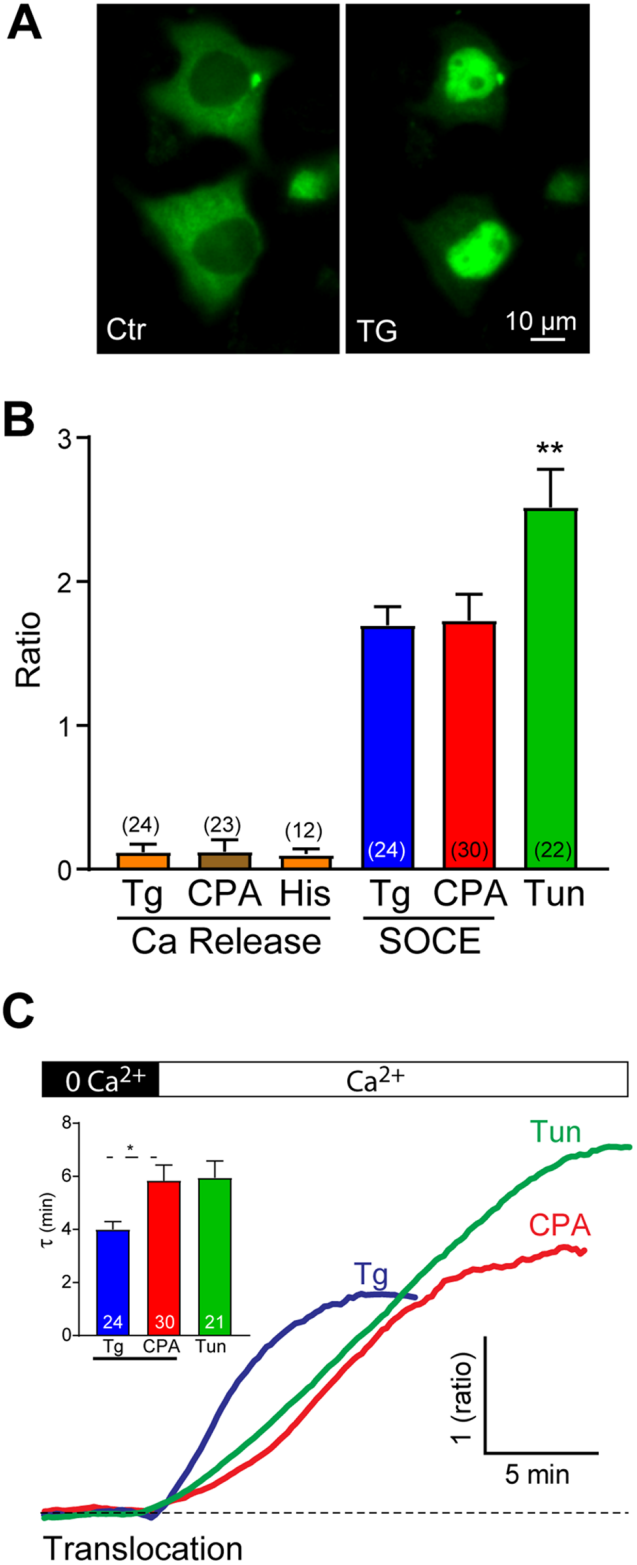
NFAT1 translocation induced by different Ca^2+^ mobilizing mechanisms. (**A**) Wide-field fluorescence images of the translocation of NFAT1 from the cytoplasm to the nucleus after store depletion with thapsigargin and activation of SOCE. (**B**) Maximum change in the nucleo-cytoplasmic ratio of NFAT1 in response to Ca^2+^ release from stores (Ca Release) using thapsigargin (Tg), CPA or histamine; or in response to Ca^2+^ influx through SOCE induced by Tg or CPA or in response to Ca^2+^ tunneling (Tun). (**C**) Example time courses of the translocation of NFAT1 induced by SOCE or by Ca^2+^ tunneling. The time constant was measured in all three conditions and summarized in a bar chart (inset). The number of experiments is indicated above or inside the bars, statistics are according to ANOVA followed by Tukey’s multiple comparison test.

A well characterized effector that responds exquisitely to Ca^2+^ in the SOCE microdomain and not Ca^2+^ release from stores is the calcineurin-NFAT signaling pathway^14,32^. NFAT1 is a transcription factor, phosphorylated at rest and dephosphorylated following the activation of calcineurin by SOCE, which leads to its translocation to the nucleus (Fig. 5A). The effect of Ca^2+^ tunneling on gK_Ca_ argues that it extends the SOCE Ca^2+^ microdomain in the cortical region of the cell and activators effectors accordingly. Therefore, Ca^2+^ tunneling should not affect the activation of calcineurin-NFAT as it is not expected to alter the SOCE microdomain. We therefore tested NFAT nuclear translocation in response to Ca^2+^ release, SOCE and Ca^2+^ tunneling (Fig. 5). Consistent with previous reports, Ca^2+^ release induced by thapsigargin, CPA or histamine did not induce NFAT1 translocation (Fig. 5B). In contrast, when SOCE was activated with either thapsigargin or CPA, it effectively induces NFAT1 translocation to the nucleus although it occurred with a faster time course in response to TG (Fig. 5A–C). Ca^2+^ tunneling results in higher levels of NFAT1 translocation with similar kinetics as those observed in response to CPA (Fig. 5C). This is likely due to the longer duration of the Ca^2+^ signal in the SOCE microdomain when Ca^2+^ tunneling is active, due to the continuous pump-leak of Ca^2+^ at the ER membrane. Alternatively, it may indicate some additional activation of calcineurin outside the SOCE microdomain in the sub-PM domain. Therefore, as expected Ca^2+^ tunneling does not dramatically alter the activation of the calcineurin-NFAT axis.

## Discussion

Agonist stimulation through GPCRs or receptor tyrosine kinases often couples to PLCs resulting in the generation of Ca^2+^ transients that in non-excitable cells tend to be biphasic, with the initial release phase due to Ca^2+^ mobilization from intracellular Ca^2+^ stores being rapid and of high amplitude but short lived as the stores empty. This is followed by a sustained phase of Ca^2+^ influx, with varying duration based on the cell type and the agonist, that is due to the activation of SOCE. Experimentally, protocols have been devised to temporally separate the Ca^2+^ release and SOCE phases as it allows for a better dissection of their relative contributions. However, physiologically the two processes are tightly linked and overlapping with SOCE being activated as stores gradually deplete and most likely in a spatially complex fashion. Therefore, in the cycle of IP_3_-dependent Ca^2+^ release, store depletion, SOCE activation, store refilling and SOCE inactivation there is a time window where SOCE is active while IP_3_ receptors are still open resulting in a pump-leak at the ER membrane, due to Ca^2+^ uptake by SERCA within the SOCE microdomain while Ca^2+^ is released through open IP_3_ receptors, a process known as Ca^2+^ tunneling. Conceptually this is reminiscent of the ‘capacitative Ca^2+^ influx’ model originally proposed by Jim Putney^33^, where he envisioned Ca^2+^ flowing directly from the extracellular space into the ER lumen to refill the stores, before the plethora of signaling roles of SOCE in addition to store refilling were appreciated. Ca^2+^ tunneling adds a new dimension for Ca^2+^ signaling downstream of SOCE by allowing Ca^2+^ influx to activate effectors that are spatially far away from the point source entry at SOCE puncta. This would be essential for SOCE to activate different effectors that do not localize to the SOCE microdomain, especially in cells such as HeLa where the SOCE puncta after store depletion are estimated to occupy <1% of the PM^12^. Furthermore, an essential feature of Ca^2+^ tunneling is to bypass the highly buffered cytosol to allow Ca^2+^ ions to reach their target by using the ER lumen as a tunnel given it’s lower buffering capacity^34^.

Ca^2+^ tunneling was originally described in pancreatic acinar cells where it transports Ca^2+^ entering at the basolateral side of the cell through ER tunnels to the apical side where IP_3_Rs localize (see Petersen *et al*. 2017 for a recent review)^2,16^. Ca^2+^ tunneling was more recently generalized through studies in frog oocytes, showing dramatic remodeling of the Ca^2+^ signaling machinery at the PM in response to store depletion to support Ca^2+^ tunneling, where it targets both Ca^2+^-activated Cl channels and the IP_3_R itself to modulate tonic versus oscillatory Ca^2+^ signaling^7,35^. The same mechanism of using ER tunnels to transport Ca^2+^ to effectors has been alluded to, although not directly investigated, in other studies where it targets the Ca^2+^-activated K channel, nuclear NFAT activation, and Ca^2+^ transport from the soma to maintain Ca^2+^ signaling in dendrites^14,30,36^.

**Figure 6:**
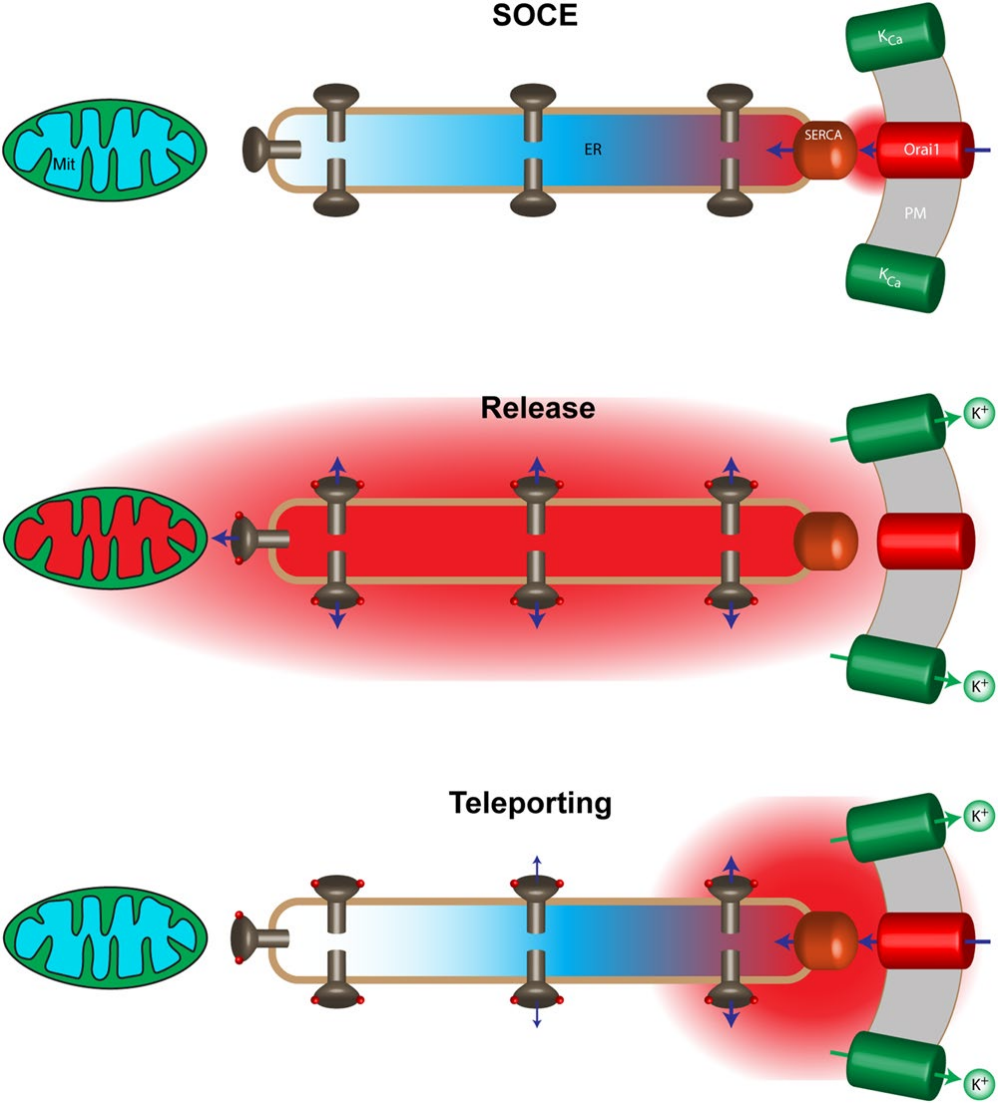
Cartoon model of the spatial distribution of Ca^2+^ signals at the SOCE microdomain, ER lumen, cytosol and mitochondria during SOCE, Ca^2+^ release on full stores, and Ca^2+^ tunneling. See text for details.

In this study, we were interested in testing the functionality of Ca^2+^ tunneling in non-polarized or specialized cells that differ from pancreatic acinar cells, neurons or oocytes for instance, using multiple different effectors of different nature with distinct spatial distribution (organelle, channel, and signaling molecule). We use simultaneous imaging of the three primary Ca^2+^ signaling compartments in HeLa cells (cytosol, ER and mitochondria) to assess the functionality of the Ca^2+^ tunneling mechanism and its ability to specifically and selectively activate downstream effectors. Our results show that Ca^2+^ tunneling is functional in HeLa cells downstream of store depletion (Fig. 2), where using combinations of agonist stimulation, manipulation of extracellular Ca^2+^, and a simple CPA-wash protocol, allows us to separate Ca^2+^ release, SOCE and Ca^2+^ tunneling to assess the effect of each Ca^2+^ signaling modules on downstream efforts. At the outset of the study an attractive target for Ca^2+^ tunneling was the mitochondria given the well documented intimate physical interaction between the ER and mitochondria through the MAMs, and their distribution away from the PM in HeLa cells^23^. Surprisingly, we show that mitochondria in HeLa cells respond readily to agonist-dependent Ca^2+^ release when stores are full with a delay after a Ca_c_^2+^ rise (Fig. 1), but do not respond to Ca^2+^ tunneling despite the fact that the Ca_c_^2+^ signal reaches a similar amplitude globally (Fig. 2). In contrast Ca^2+^ tunneling was more effective than Ca^2+^ release and far more effective than SOCE at activating gK_Ca_ and at raising sub-PM Ca^2+^ levels. Mitochondria in HeLa cells do not respond well to Ca^2+^ flowing through SOCE^23^, a finding that we have confirmed here (Fig. 1). However, the mitochondrial response to Ca^2+^ tunneling is even poorer than to maximal SOCE stimulated by thapsigargin (Fig. 2B). The chain of channels and pumps mediating Ca^2+^ tunneling in series could potentially explain this observation. Ca^2+^ influx through SOCE initiates Ca^2+^ tunneling followed by Ca^2+^ uptake into the depleted ER through SERCA activity, and then release through open IP_3_Rs. The limiting factor as discussed below in this Ca^2+^ transport chain is the SERCA pump given its low flux. Estimates of the single channel Ca^2+^ current through the IP_3_R under physiological conditions are 0.1-0.2 pA^37^, corresponding to a rate of 6 × 10^5^ Ca^2+^/sec at the lower end of the spectrum. The rate of SERCA2b uptake was estimated at ~40 Ca^2+^/sec at Vmax^6,38^, and the flux through an Orai channel is estimated at 5000 Ca^2+^/sec considering a P_o_ of 0.8 (see Hogan for a detailed discussion)^6^. These estimates argue that during Ca^2+^ tunneling the leak through any open IP_3_R is orders of magnitude higher than the trickle of Ca^2+^ that can be fed into the ER by SERCA. This would ensure that the ER does not refill readily allowing Ca^2+^ tunneling to proceed for tens of seconds. Assuming that IP_3_Rs are evenly distributed throughout the ER membrane and opening stochastically the first open IP_3_R encountered would release the Ca^2+^ fed into the ER by 15,000 SERCA pumps operating at full capacity upstream. This implies that during tunneling Ca^2+^ entering the cell through SOCE would not be able to travel deep within the cell as the ER acts as a sieve with open IP_3_Rs resulting in Ca^2+^ leak from the cortical ER, thus preventing Ca^2+^ from reaching deep within the ER to activate mitochondria (Fig. 6). This model is attractive, as it would also explain the differential response of gKca and mitochondria to Ca^2+^ tunneling (Fig. 6). The remodeling of the Ca^2+^ signaling machinery in response to store depletion results in SOCE as a point source Ca^2+^ entry that localizes to focal sites at the PM and as such directionally feeding Ca^2+^ into the cell. At this point, we cannot rule out other contributing factors such as the slower speed of Ca^2+^ tunneling as compared to Ca^2+^ release or some kind of restructuring to the MAMs (Supplemental Fig. 3).

The flux estimates discussed above would also predict leakage of Ca^2+^ from the SOCE microdomain since the flow of Ca^2+^ through Orai channels in SOCE puncta would be predicted to overwhelm SERCA pumps that localize to the puncta. Consistently, we observe an increase in sub-PM Ca^2+^ and activation of gKca in response to SOCE. However, Ca^2+^ tunneling greatly enhance both as would be expected from the leak through IP_3_Rs (Fig. 6). Furthermore, Ca^2+^ tunneling by maintaining the ER depleted extends the duration of SOCE and allows it to more effectively activate downstream effectors. As shown in Fig. 3, although the amplitude of gK_Ca_ induced by Ca^2+^ tunneling is much smaller than that induced by Ca^2+^ release, the total charge transfer is significantly greater in response to Ca^2+^ tunneling. This is reflected as well in the Ca^2+^ signal in the sub-PM domain (Fig. 4).

NFAT activation in contrast to gK_Ca_ or the mitochondrial response, is quite specific to a Ca^2+^ rise in the SOCE microdomain as it is activated with equal efficiency whether SOCE is induced maximally using thapsigargin or to physiological levels using the CPA-wash protocol (Fig. 5). This is consistent with previous studies^14,32,39^. There is a statistically significant increase in NFAT translocation when Ca^2+^ tunneling is activated (Fig. 5B). This could be due to either the increased duration of the Ca^2+^ signal in the SOCE microdomain due to Ca^2+^ tunneling (Fig. 4B) or alternatively activation of calcineurin that localizes outside the SOCE clusters that can be targeted by Ca^2+^ tunneling.

Collectively, our data show that Ca^2+^ tunneling is functional in HeLa cells and expands the specificity of the Ca^2+^ signaling machinery toward downstream effectors and sub-cellular domains. The response of the mitochondria and gK_Ca_ shows that Ca^2+^ tunneling is particularly effective at raising Ca^2+^ levels in the cortical cytoplasm next to the PM. This can be explained by the high conductance of IP_3_Rs as discussed above, leading to a strong leak that favors Ca^2+^ release in the cortical region close to the SOCE point source Ca^2+^ entry. It therefore appears that Ca^2+^ tunneling is a specialized module to raise cortical Ca^2+^ levels thus effectively expanding the SOCE microdomain. This is somewhat distinct from the long range Ca^2+^ transport due to tunneling in pancreatic acinar cells, which supports the transport of Ca^2+^ through the ER lumen from the basolateral to the luminal end of the cell. Ca^2+^ tunneling in addition to expanding the spatial spread of the SOCE microdomain also modulates the temporal dynamics of SOCE by extending the duration of SOCE by maintaining a depleted ER and lower Ca^2+^ levels in the SOCE microdomain due to the continuous pumping of Ca^2+^ into the ER lumen by SERCA and its release through IP_3_Rs (Fig. 6). Finally, and consistent with our findings, Thillaiappan *et al*. recently showed that licensed IP_3_Rs preferentially localize close to the PM in the cell cortex and at the periphery of STIM1 clusters^40^. Such a localization of immobile IP_3_Rs is ideally suited to support Ca^2+^ tunneling and in particular providing it with its cortical spatial specificity as discussed herein.

## Methods

### Cell culture and solutions

Hela cells were cultured in DMEM media containing 10% fetal bovine serum supplemented with penicillin (100 units.ml^−1^) and streptomycin (100 µg.ml^−1^). The cells were plated 24 h before transfection on poly-lysine coated glass-bottom dishes (MatTek, U.S.A). For all live cells experiments, the cells were continuously perfused using a peristaltic pump (Gilson Minipuls) at the speed of 1 ml.min^−1^. The standard saline contained (in mM) 145 NaCl, 5 KCl, 2 CaCl_2_, 1 MgCl_2_, 10 Glucose, 10 HEPES, pH 7.2, for Ca^2+^-free experiments, the Ca^2+^ was exchanged equimolarly with Mg^2+^.

### Intracellular Ca^2+^ imaging

To perform organelle Ca^2+^ imaging HeLa cells were transfected with the Ca^2+^ indicators G-CEPIA2mt and R-CEPIAer constructs (0.5 µg per dish) using a standard lipofectamine 2000 (Invitrogen) procedure 24 h to 48 h prior to imaging. Both constructs were obtained from addgene (#58218 and #58216 respectively) and were originally created by Masamitsu Lino’s group^25^. To image cytoplasmic Ca^2+^ the cells were loaded for 45 min with Fura Red-AM at 37 °C (1 µM from a 1 mM stock in 20% pluronic acid/DMSO). Imaging was performed on a Leica TCS SP5 confocal system (Leica, Germany) fitted with a 63x/1.4-06 oil immersion objective using an open pinhole. The G-CEPIA2mt was excited using a 488 nm laser line and the emission collected at 500–590 nm. The same line was used to excite the Ca^2+^-free form of FuraRed and the emission collected between 600–709 nm. For R-CEPIAer the excitation was performed with a 561 nm laser line and the signal collected between 583–649 nm. The frame rate was set to 0.1 Hz unless stated otherwise. For Fluo4 imaging, the cells were loaded with 1–2 µM Fluo4AM (45 min/37 °C). The excitation was performed using a 488 nm laser line and the signal collected at 500–560 nm with the pinhole at 1 airy unit and the frame rate was set to 1 Hz.

### Morphological analysis

Cells expressing G-CEPIA2mt and R-CEPIAer were fixed (PFA 4%, 10 min) and stained with Alexa633 tagged Wheat Germ Agglutinin (2 µg.ml^−1^) (Invitrogen). Confocal images were acquired every 0.5 µm to generate z-stacks. The imaging was performed on a Zeiss LSM880 controlled by Zen Black 2.3 (Zeiss, Germany) and fitted with a 63x/1.4 objective. The imaging parameters were as follows: for G-CEPIA2mt: λ_ex_ = 488 nm, λ_em_ = 494–568 nm, for R-CEPIAer: λ_ex_ = 561 nm, λ_em_ = 570–622 nm and for WGA-Ax633: λ_ex_ = 633 nm, λ_em_ = 640–747 nm. The distance between the plasma membrane, the ER and the mitochondria was evaluated using the profile of a linear region of interest drawn between both sides of the cell and the distance measured at 50% of the peak amplitudes of the signals.

### Whole-cell patch-clamp

The Ca^2+^ activated K^+^ channels were recorded using a standard whole-cell patch-clamp protocol. Patch pipettes (resistance ranging from 4 to 6 MΩ when filled with the pipette solution) were sealed to the plasma membrane and the patch ruptured after the formation of a giga-ohm seal. The cells were voltage-clamped at 0 mV at steady state using an Axopatch 200B amplifier (Molecular Devices, U.S.A) controlled by pClamp 10. The internal pipette solution contained (in mM) 140 K-Gluconate, 2 NaATP, 2 MgCl_2_, 10 HEPES, 1 µM EGTA and pH 7.4. The extracellular solutions and perfusion system was the same as the imaging experiments.

### Total Internal Reflection Fluorescence Microscopy (TIRF)

For the localization at the plasma membrane, the cells where transfected with the Orai1-mCh, STIM1-CFP and CEPIA2mt constructs, and the stores depleted with thapsigargin. The membrane plane was identified by the presence of clusters of STIM1 and Orai1, and used to adjust the evanescent wave. The imaging was performed on a Zeiss Cell observer TIRF system using the following parameters for STIM1-CFP: λ_ex_ = 405 nm, λ_em_ = 446–468 nm, for G-CEPIA2mt: λ_ex_ = 488 nm, λ_em_ = 510–555 nm and for Orai1-mCh: λ_ex_ = 561, λ_em_ = 581–679 nm. For TIRF Ca^2+^ imaging at the plasma membrane, the cells were transfected with the Ca^2+^ sensor Lck-GCamp5G (Addgene #34924)^31^. The sensor was excited at λ_ex_ = 488 nm and the images collected using λ_em_ = 510–555 nm, the frame rate was 0.1 Hz. The perfusion system and solutions was identical to the previous experiments.

### NFAT1 translocation

Cells were transfected for 24 h with the NFAT1-GFP construct (Addgene #11107)^41^, the imaging was performed using the same settings as the TIRF imaging for GFP-tagged proteins except that the mirror was set vertically to obtain a widefield image. The perfusion saline and system were as previously described.

### Data analysis and statistics

The imaging data was quantified using FIJI/ImageJ 1.51 n^42,43^ and ZenBlue 2.3 (Zeiss). The patch-clamp data was analyzed with Clampfit 10.0 (Molecular Devices). Statistics and data analysis were performed using Graphpad Prism 7.02 (GraphPad U.S.A). Values are given as means ± S.E.M and statistics were performed using either paired or unpaired Student’s t-test or ANOVA followed by Tukey’s test for multiple comparisons. P-values are ranked as follows *P < 0.05, **P < 0.01, ***P < 0.001.

## Electronic supplementary material

Supplementary Information

## References

[1] Filadi R, Basso E, Lefkimmiatis K, Pozzan T (2017). Beyond Intracellular Signaling: The Ins and Outs of Second Messengers Microdomains. Adv Exp Med Biol.

[2] Petersen OH, Courjaret R, Machaca K (2017). Ca2+ tunnelling through the ER lumen as a mechanism for delivering Ca2+ entering via store-operated Ca2+ channels to specific target sites. J Physiol.

[3] Prakriya M, Lewis RS (2015). Store-Operated Calcium Channels. Physiol Rev.

[4] Frischauf I, Fahrner M, Jardin I, Romanin C (2016). The STIM1: Orai Interaction. Adv Exp Med Biol.

[5] Elaib Z, Saller F, Bobe R (2016). The Calcium Entry-Calcium Refilling Coupling. Adv Exp Med Biol.

[6] Hogan PG (2015). The STIM1-ORAI1 microdomain. Cell Calcium.

[7] Courjaret R, Machaca K (2014). Mid-range Ca2+ signalling mediated by functional coupling between store-operated Ca2+ entry and IP3-dependent Ca2+ release. Nature communications.

[8] Sampieri A, Zepeda A, Asanov A, Vaca L (2009). Visualizing the store-operated channel complex assembly in real time: identification of SERCA2 as a new member. Cell Calcium.

[9] Jousset H, Frieden M, Demaurex N (2007). STIM1 knockdown reveals that store-operated Ca2+ channels located close to sarco/endoplasmic Ca2+ ATPases (SERCA) pumps silently refill the endoplasmic reticulum. J. Biol Chem..

[10] Manjarres IM, Rodriguez-Garcia A, Alonso MT, Garcia-Sancho J (2010). The sarco/endoplasmic reticulum Ca(2+) ATPase (SERCA) is the third element in capacitative calcium entry. Cell Calcium.

[11] Wu MM, Buchanan J, Luik RM, Lewis RS (2006). Ca2+ store depletion causes STIM1 to accumulate in ER regions closely associated with the plasma membrane. Journal of Cell Biology.

[12] Orci L (2009). STIM1-induced precortical and cortical subdomains of the endoplasmic reticulum. Proc. Natl. Acad. Sci. USA.

[13] Chang CL, Chen YJ, Liou J (2017). ER-Plasma Membrane Junctions: Why and How Do We Study Them?. Biochim Biophys Acta.

[14] Kar P, Mirams GR, Christian HC, Parekh AB (2016). Control of NFAT Isoform Activation and NFAT-Dependent Gene Expression through Two Coincident and Spatially Segregated Intracellular Ca2+ Signals. Mol Cell.

[15] Willoughby D (2012). Direct binding between Orai1 and AC8 mediates dynamic interplay between Ca2+ and cAMP signaling. Sci Signal.

[16] Mogami H, Nakano K, Tepikin AV, Petersen OH (1997). Ca2+ flow via tunnels in polarized cells: recharging of apical Ca2+ stores by focal Ca2+ entry through the basal membrane patch. Cell.

[17] Raturi A, Simmen T (2013). Where the endoplasmic reticulum and the mitochondrion tie the knot: the mitochondria-associated membrane (MAM). Biochim Biophys Acta.

[18] Hoth M, Button DC, Lewis RS (2000). Mitochondrial control of calcium-channel gating: a mechanism for sustained signaling and transcriptional activation in T lymphocytes. Proc. Natl. Acad. Sci. USA.

[19] Hoth M, Fanger CM, Lewis RS (1997). Mitochondrial regulation of store-operated calcium signaling in T lymphocytes. Journal of Cell Biology.

[20] Glitsch MD, Bakowski D, Parekh AB (2002). Store-operated Ca2+ entry depends on mitochondrial Ca2+ uptake. EMBO J.

[21] Carafoli E (2012). The interplay of mitochondria with calcium: an historical appraisal. Cell Calcium.

[22] Park MK, Ashby MC, Erdemli G, Petersen OH, Tepikin AV (2001). Perinuclear, perigranular and sub-plasmalemmal mitochondria have distinct functions in the regulation of cellular calcium transport. EMBO J.

[23] Giacomello M (2010). Ca2+ hot spots on the mitochondrial surface are generated by Ca2+ mobilization from stores, but not by activation of store-operated Ca2+ channels. Mol Cell.

[24] Lawrie AM, Rizzuto R, Pozzan T, Simpson AW (1996). A role for calcium influx in the regulation of mitochondrial calcium in endothelial cells. J Biol Chem.

[25] Suzuki J (2014). Imaging intraorganellar Ca2+ at subcellular resolution using CEPIA. Nature communications.

[26] Patron M (2014). MICU1 and MICU2 finely tune the mitochondrial Ca2+ uniporter by exerting opposite effects on MCU activity. Mol Cell.

[27] English AR, Voeltz GK (2013). Endoplasmic reticulum structure and interconnections with other organelles. Cold Spring Harb Perspect Biol.

[28] Collins TJ, Lipp P, Berridge MJ, Bootman MD (2001). Mitochondrial Ca(2+) uptake depends on the spatial and temporal profile of cytosolic Ca(2+) signals. Journal of Biological Chemistry.

[29] Diarra A, Wang R, Garneau L, Gallo-Payet N, Sauve R (1994). Histamine-evoked Ca2+ oscillations in HeLa cells are sensitive to methylxanthines but insensitive to ryanodine. Pflugers Arch.

[30] Liu X, Rojas E, Ambudkar IS (1998). Regulation of KCa current by store-operated Ca2+ influx depends on internal Ca2+ release in HSG cells. The American journal of physiology.

[31] Akerboom J (2012). Optimization of a GCaMP calcium indicator for neural activity imaging. J Neurosci.

[32] Kar P, Parekh AB (2015). Distinct spatial Ca2+ signatures selectively activate different NFAT transcription factor isoforms. Mol Cell.

[33] Putney JW (1986). A model for receptor-regulated calcium entry. Cell Calcium.

[34] Wu X, Bers DM (2006). Sarcoplasmic reticulum and nuclear envelope are one highly interconnected Ca2+ store throughout cardiac myocyte. Circ Res.

[35] Courjaret R, Dib M, Machaca K (2017). Store-Operated Ca2+ Entry in Oocytes Modulate the Dynamics of IP3 -Dependent Ca2+ Release From Oscillatory to Tonic. J Cell Physiol.

[36] Choi YM, Kim SH, Chung S, Uhm DY, Park MK (2006). Regional interaction of endoplasmic reticulum Ca2+ signals between soma and dendrites through rapid luminal Ca2+ diffusion. J Neurosci.

[37] Foskett JK, White C, Cheung KH, Mak DO (2007). Inositol trisphosphate receptor Ca2+ release channels. Physiol Rev..

[38] Lytton J, Westlin M, Burk SE, Shull GE, MacLennan DH (1992). Functional comparisons between isoforms of the sarcoplasmic or endoplasmic reticulum family of calcium pumps. J Biol Chem.

[39] Kar P (2014). Dynamic assembly of a membrane signaling complex enables selective activation of NFAT by orai1. Current biology: CB.

[40] Thillaiappan NB, Chavda AP, Tovey SC, Prole DL, Taylor CW (2017). Ca(2+) signals initiate at immobile IP3 receptors adjacent to ER-plasma membrane junctions. Nature communications.

[41] Aramburu J (1999). Affinity-driven peptide selection of an NFAT inhibitor more selective than cyclosporin. A. Science.

[42] Schindelin J (2012). Fiji: an open-source platform for biological-image analysis. Nat Methods.

[43] Schneider CA, Rasband WS, Eliceiri KW (2012). NIH Image to ImageJ: 25 years of image analysis. Nat Methods.

