# Supplementary material for: Spatially restricted subcellular Ca2+ signaling downstream of store-operated calcium entry encoded by a cortical tunneling mechanism
Source: Sci Rep. 2018 Jul 25;8:11214. doi: 10.1038/s41598-018-29562-9 (PMC6060099; doi:10.1038/s41598-018-29562-9)
Supplement: Supplementary file 1 — Supplementary Information [file 41598_2018_29562_MOESM1_ESM.docx]

**SREP-18-09709A**

**Title: Spatially restricted subcellular Ca^2+^ signaling downstream of store-operated**

**calcium entry encoded by a cortical tunneling mechanism**

Authors: Raphael Courjaret, Maya Dib and Khaled Machaca

**Supplemental Figures**

***Supplemental Figure 1: Kinetics of Ca^2+^ release on full stores and during Ca^2+^ tunneling.***

Relative time courses of Ca^2+^ changes in the cytosol (purple) and in the mitochondria (green) induced by histamine application and recorded at a speed of 1 Hz

***Supplemental Figure 2: Effect of a high extracellular Ca^2+^ concentration on Ca^2+^ tunneling.***

(**A**) Live recording of Ca^2+^ levels in the cytosol (Cyt) using Fura-red, in the ER using the CEPIAER reporter and in the mitochondria (Mit) using CEPIA2mt. The stores were depleted using CPA and SERCA function restored by washing out the inhibitor. Histamine (His) is applied together with a high Ca^2+^ (10 mM). This increase in the driving force for Ca^2+^ ions did not induce a Ca^2+^ rise in mitochondria in response to Ca^2+^ tunneling. (**B**) Bar charts summarizing the effect of a high extracellular Ca^2+^ concentration on Ca^2+^ in the cytosol and the mitochondria as indicated, during tunneling (Tun) and during a normal release event (Rel). The cytosolic signal was not increased significantly by the high Ca^2+^, arguing that Ca^2+^ flow through SOCE is not the limiting factor preventing the signal to reach the mitochondria.

***Supplemental Figure 3: Kinetics of Ca^2+^ release on full stores and during Ca^2+^ tunneling.***

Cells were loaded with Fluo4-AM and imaged at a scanning speed of 1Hz. (A) Confocal images obtained during Ca^2+^ tunneling when histamine is applied on depleted stores using SOCE as the Ca^2+^ source and when the stores are refilled. (B) Kinetics of the Ca^2+^ release obtained using lines as illustrated in (A), the peak is clearly faster when the stores are full *vs* empty. (C) Average time course of the cytosolic Ca^2+^ release induced by histamine on depleted stores (blue, Tun) and after refilling (red, Rel). (D) The rising phase of the Ca^2+^ signal was fitted with a linear regression from 2080 % of the peak and the slope compared between empty and full stores.

***Supplemental Figure 4: Localization of the ER and mitochondria relative to the plasma membrane.***

(A) Cells expressing G-CEPIA2mt (green) and R-CEPIAer (red) were fixed and stained with wheat germ agglutinin coupled to Alexa633 (purple) to reveal the plasma membrane (PM). (B) Orthogonal sections reconstructed from z-stacks of confocal optical slices as in (A). (C) Intensity of the signal for the mitochondria, ER and plasma membrane along lines traced as indicated in (A) before and after store depletion with thapsigargin. (D) Quantification of the distance between the PM and the mitochondria taken at 50 % of the maximum value before and after store depletion. (E) Imaging of the signal in wide-field (WF) and in the TIRF plane from cells expressing the SOCE partners STIM1-CFP (blue) and Orai1-mCherry (red) as well as the G-CEPIA2mt (green). Stores were depleted with thapsigargin. The absence of a green signal in the TIRF plane suggests low abundance of mitochondria near the PM. (F) Cartoon depicting the analysis in the zaxis of the relative positions of the ER, the mitochondria and the plasma membrane. The intensities in a disc 5 µm in diameter were analyzed along the vertical axis of the cell. (G) Intensity plots along the z-axis of the cell before and after store depletion with thapsigargin. The average distance between the first mitochondria and the plasma membrane is represented in a bar chart (inset). Values are means ± S.E.M., the number of measures is indicated in the bars or in the graphs.

Statistics are according to Student’s unpaired t-test.

***Supplemental Figure 5: Morphology of the mitochondria after store depletion by CPA.***

Cells expressing G-CEPIA2mt have been imaged using a Confocal Microscope equipped with an Airy Scan detector and controlled by Zen 2.3 (Zeiss). The lens was a 63x/1.4 and the fast Airy Scan function of the sensor in super-resolution mode was used. Z-stacks were obtained at a spacing of 0.19μm (optimal) and the images processed using the internal routine of the Zen software. The analysis of the images were performed using ImageJ 1.52b^1^ using the “BoneJ” plugin^2^. The complete z-stacks were thresholded using the built in automatic function of ImageJ, then the mitochondria skeleton was extracted from the image and analysed using BoneJ. The average branch length was plotted before and after perfusion of CPA.

(A) 3D reconstruction of the morphology of the mitochondria in HeLa cells before (Ctr) and after store depletion with CPA. The 3D reconstruction was performed using Zen 2.3. (B) Example of the skeleton extracted from the image in (A) using the BoneJ plugin. (C) Comparison of the average lengths of the branches of the mitochondria measured before (Ctr), after store depletion (CPA) and after refilling the stores using a Ca^2+^-containing extracellular solution. Although there was a small significant reduction in the branch lengths during store depletion, it could not be reversed when refilling the stores. Statistics according to ANOVA followed by Tukey’s multiple comparison test, the number of experiments is indicated in the green bar.

***Supplemental Figure 6: Ionic currents activated by Ca^2+^ release from the ER***

Whole cell patch-clamp was performed in the conditions indicated in the main text. To evaluate the current/voltage relationship of the current the cells were held at a holding potential of -50 mV and voltage ramps from -100 mV to +100 mV (1s in duration, 0.1 Hz in frequency were applied). (A) Current voltage relationship of the global outward current recorded before (0s) and during the application of histamine (His, 100 μM, 30s in duration). (B) Time course illustrating the development of the current induced by histamine and reported in (A) at 0 mV during the voltage ramp. (C) Current-voltage relationships obtained before (0s) during (10s) and after (60 s and 80s) the application of histamine on HeLa cells. The values are averaged from 4 cells. (D) Outward currents induced by the application of thapsigargin (1 μM, 10 min in a Ca^2+^-free media) to depleted intracellular Ca^2+^ stores and by the activation of SOCE when Ca^2+^ is re-added in the extracellular media.


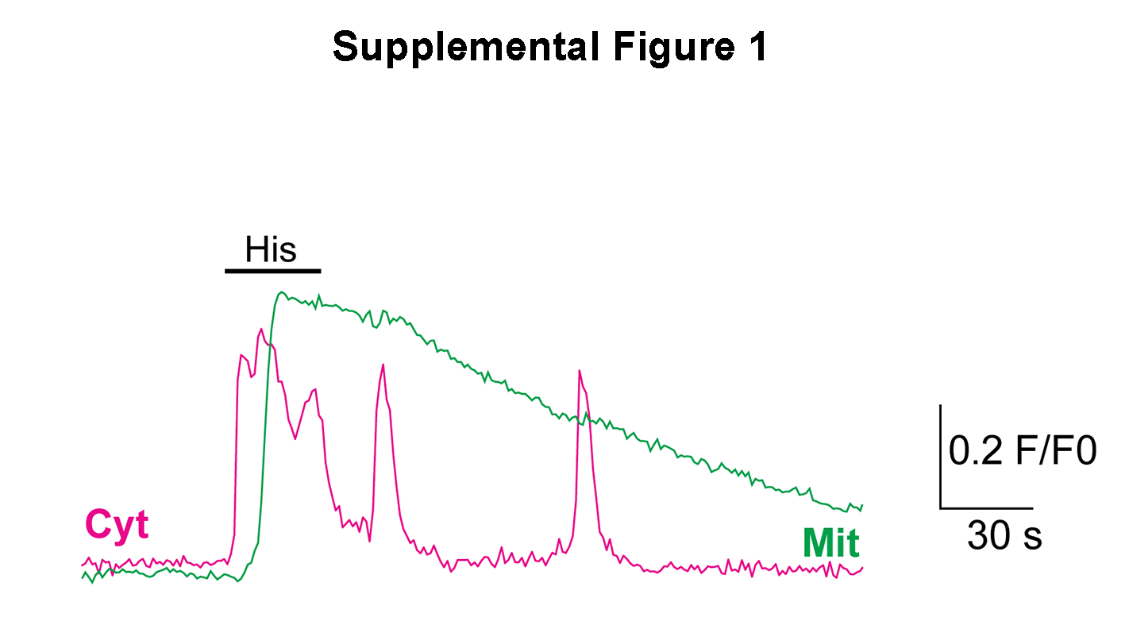


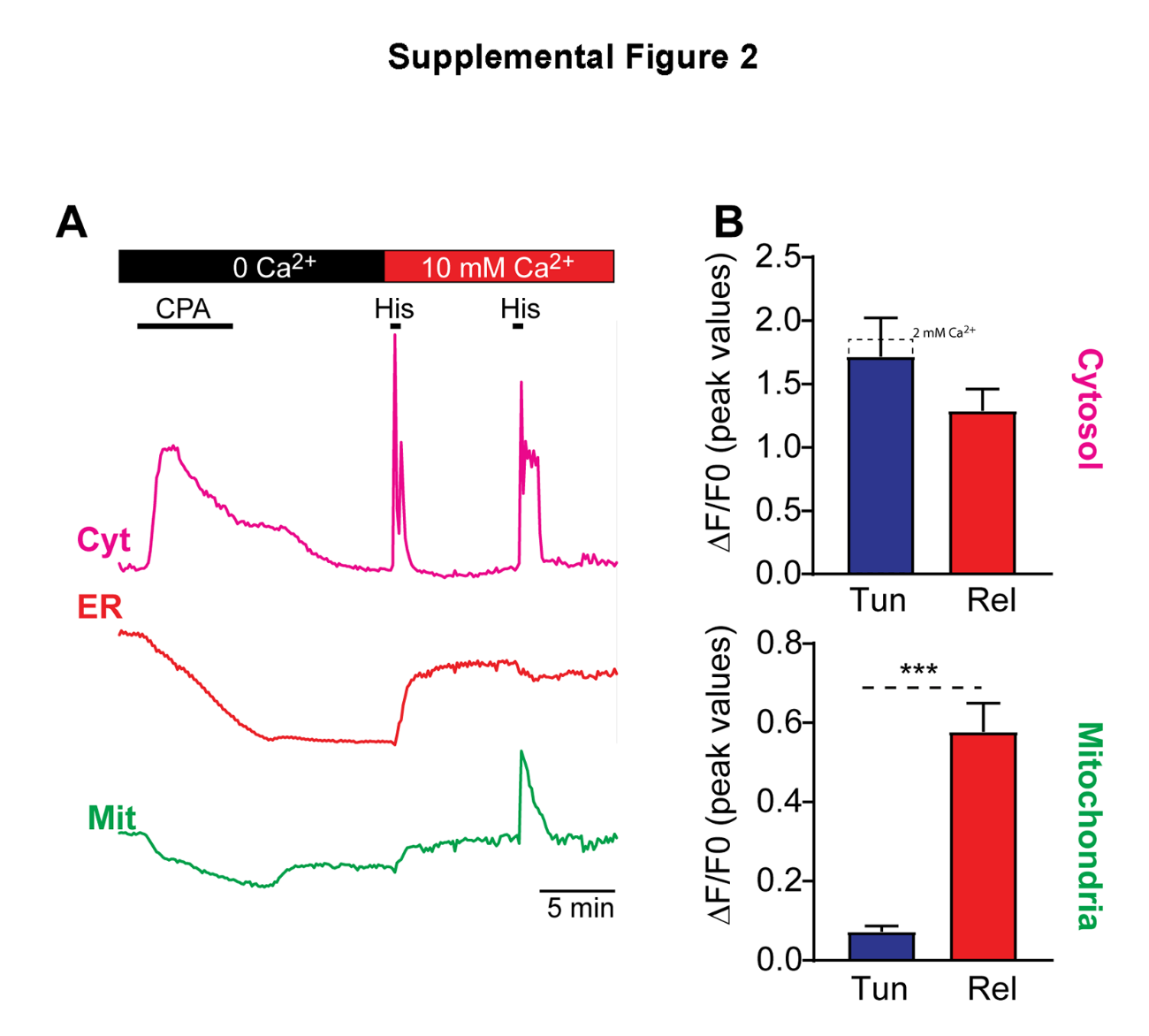


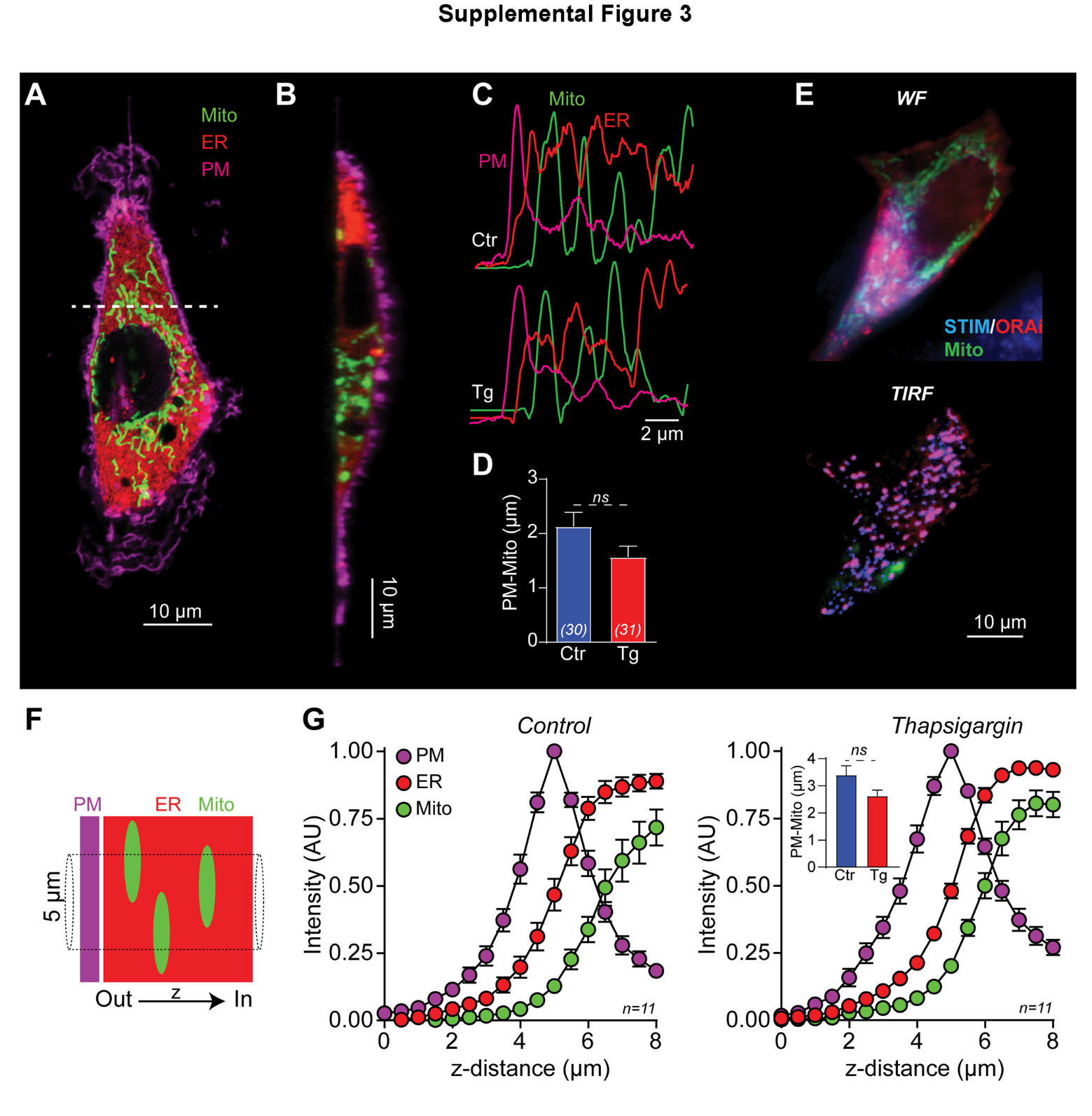


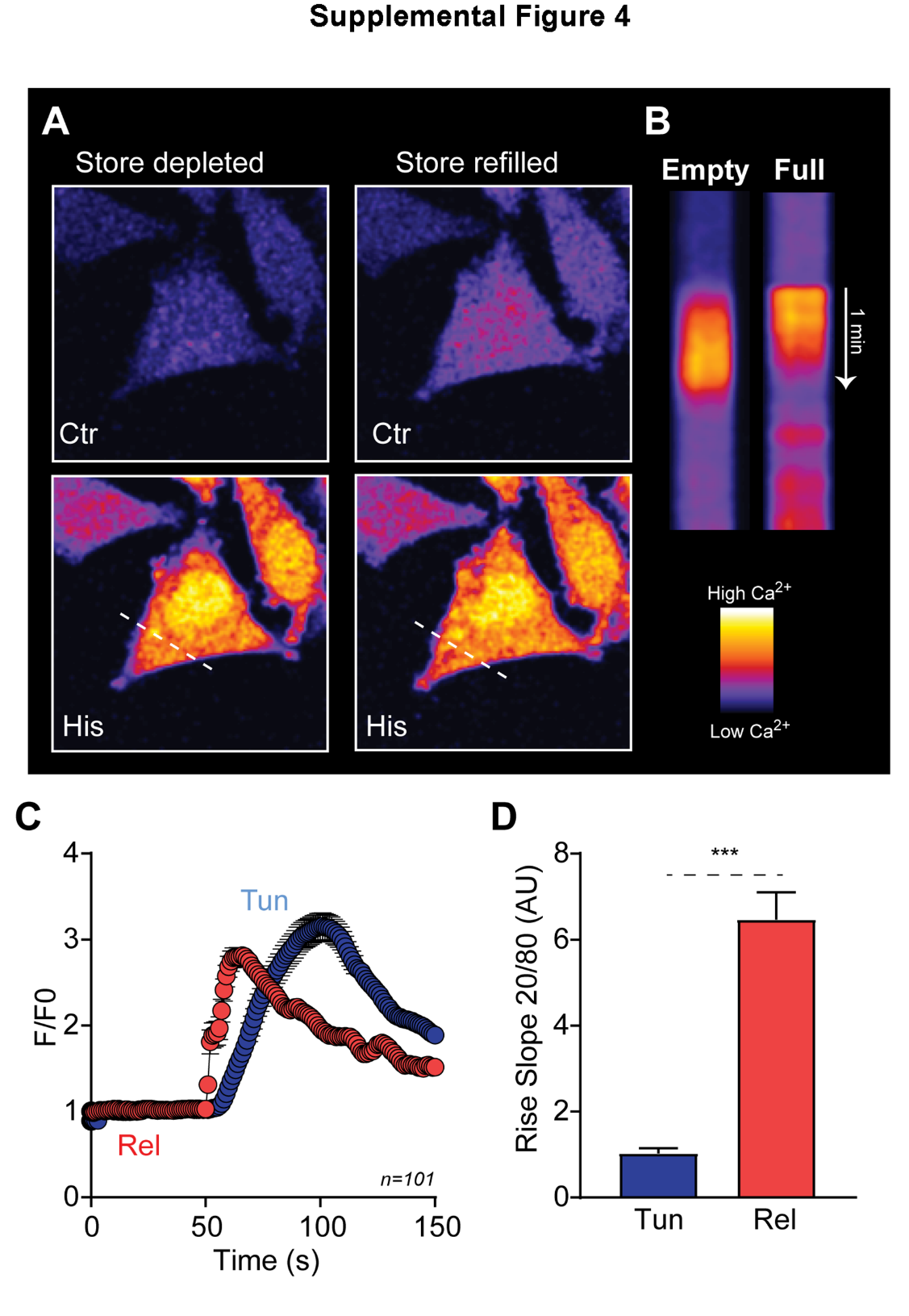


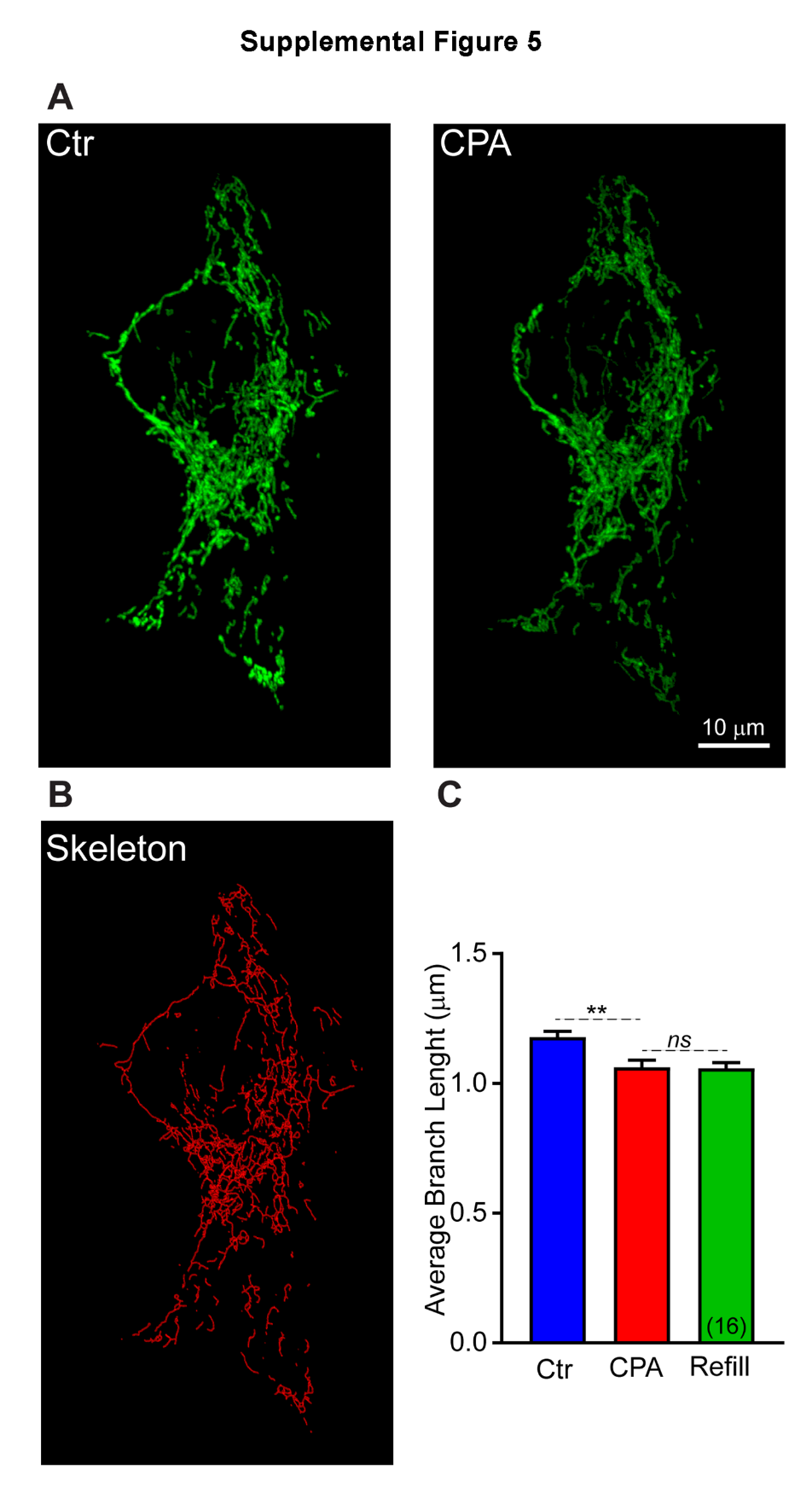


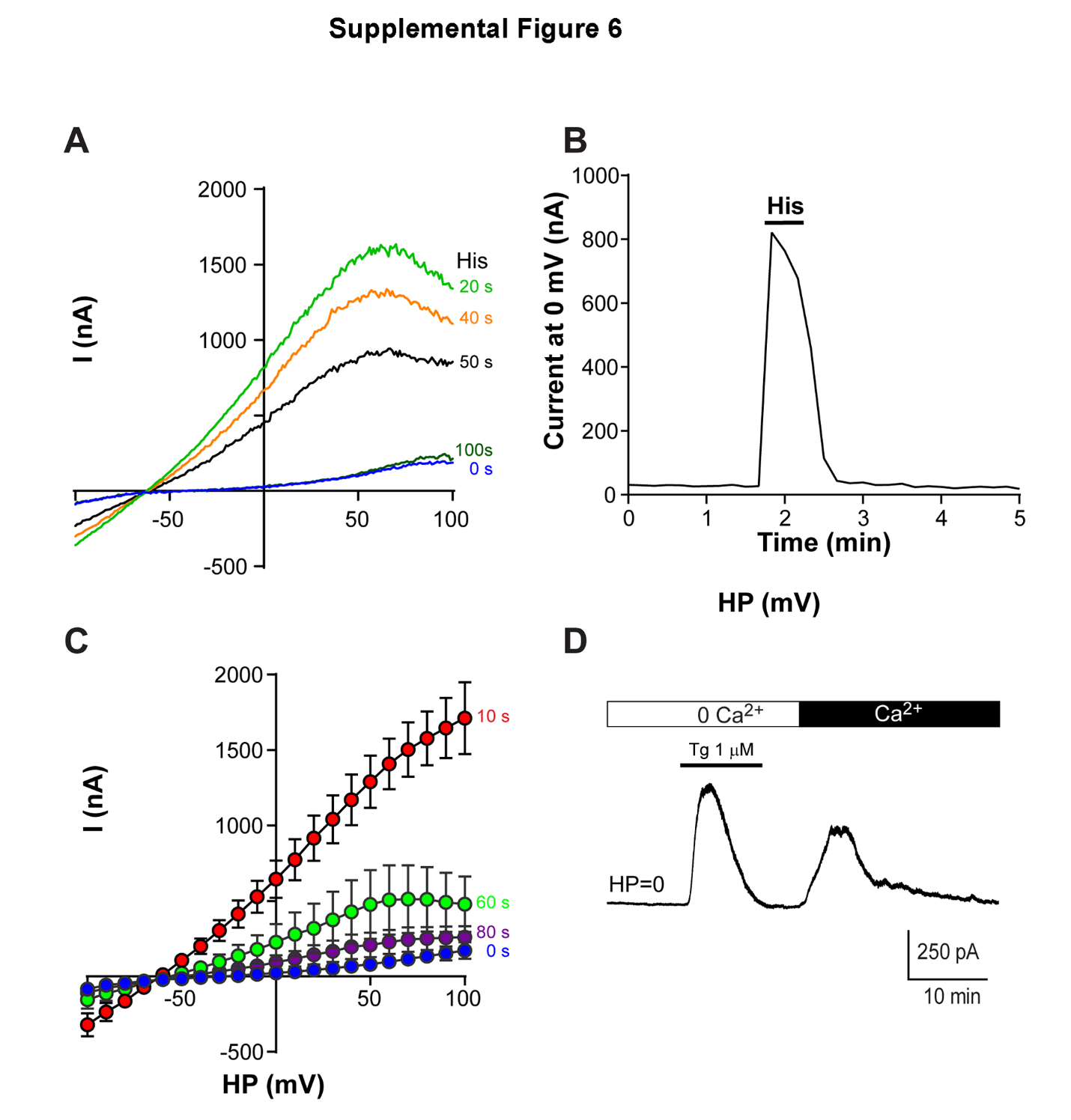


**Supplemental References**

1 Schneider, C. A., Rasband, W. S. & Eliceiri, K. W. NIH Image to ImageJ: 25 years of image analysis. *Nat Methods* **9**, 671-675 (2012).

2 Doube, M. *et al.* BoneJ: Free and extensible bone image analysis in ImageJ. *Bone* **47**, 1076-1079, doi:10.1016/j.bone.2010.08.023 (2010).
